# ASSOCIATIONS BETWEEN GAIT MEASURES AND QUADRICEPS MUSCLE THICKNESS AS MEASURED BY POINT-OF-CARE ULTRASOUND

**DOI:** 10.1093/geroni/igad104.2927

**Published:** 2023-12-21

**Authors:** Uyanga Ganbat, Boris Feldman, Shane Arishenkoff, Graydon Meneilly, Kenneth Madden

**Affiliations:** University of British Columbia, Vancouver, British Columbia, Canada; University of British Columbia, Vancouver, British Columbia, Canada; Vancouver General Hospital, Vancouver, British Columbia, Canada; University of British Columbia, Vancouver, British Columbia, Canada; University of British Columbia, Vancouver, British Columbia, Canada

## Abstract

Gait parameters and sarcopenia predict falls risk which is one of the major causes of both mortality and morbidity among older adults. Our objective was to evaluate whether anterior thigh muscle measured by point-of-care ultrasound is significantly associated with standard gait measures. All subjects were referred from ambulatory geriatric medicine clinics at an academic center. Quadriceps muscle thickness was measured by a portable ultrasound device. Gait variables were measured by the patient walking for 6 minutes. The primary response variables were gait variables, and the predictor variables were age, biological sex, body mass index, and MT. Univariate and multivariate regression analyses were performed. A total of 150 participants were recruited from geriatric medicine clinics. Muscle thickness was measured in 149 participants and the mean (SD) was 1.91 (0.52) (median 1.82 cm, 0.96 to 3.68 cm). Among all the gait variables, average swing time (P = 0.010) and average stance time (P = 0.010) were correlated significantly with muscle thickness. Muscle thickness also showed a negative association with step time variability percent (P = 0.005). Muscle thickness (P = 0.046), age (P = 0.025), and gender (P = 0.047) had a statistically significant association with present step time variability. PoCUS showed a significant association with average swing time, average stance time, and step time variability all of which are predictive of future falls risk. Although more work needs to be done, PoCUS is a reliable and feasible muscle measurement method, which could lead to faster clinical decision-making and falls risk assessment.

